# Continuing Efforts in Global Chronic Disease Prevention

**Published:** 2007-03-15

**Authors:** David V McQueen

**Affiliations:** National Center for Chronic Disease Prevention and Health Promotion, Centers for Disease Control and Prevention

This issue of *Preventing Chronic Disease* (*PCD*) illustrates some of the breadth of work in chronic disease prevention being undertaken throughout the world. The wide range of activity includes health promotion, descriptive epidemiology, behavioral risk-factor surveillance, and purely exploratory and descriptive research. The emphasis of this issue is on efforts being made in economically less-developed parts of the world as well as on efforts being made to address the needs of subgroups within more economically advanced countries.

Chronic diseases (usually termed noncommunicable diseases in the international health literature) are generally characterized by a long latency period, a mixture of causal factors including some well-known risk factors, a prolonged course of illness, a noncontagious origin, functional impairment or disability, and incurability. In addition, many globally important communicable diseases (e.g., AIDS, polio) have chronic characteristics. The global cost of these conditions, in human as well as in financial terms, is enormous, and the burden that they impose is especially critical in countries that are economically less well off.

By the last decade of the 20th century, chronic diseases had superseded communicable diseases as the leading cause of death in all areas of the world except sub-Saharan Africa and the Middle East, and within the next 15 years, chronic diseases are projected to account for nearly three quarters of all deaths in low-income regions of the world (1).

Two points about the impact of chronic diseases are important to keep in mind. First, because around 80% of the world's population lives in industrializing or nonindustrialized countries, these countries will experience most of the cases of death and disability associated with major chronic diseases, and such widespread disability and death may have catastrophic effects on the health care infrastructure and the further economic development of many of them. Second, the relatively poor quality of health care services available to lower-income populations, in both developing and highly developed countries, continues to exacerbate the increased risk for chronic health problems associated with such key factors as urbanization and an aging population. In addition, the worldwide increase in average lifespan has also contributed to the increased global threat posed by chronic diseases. For low-income countries, lower birth rates coupled with greater life expectancy and an increased risk for chronic diseases portend dramatic increases in both the relative and absolute importance of chronic conditions such as ischemic heart disease, stroke, diabetes, and depression.

Most chronic diseases are associated with or caused by a combination of social, cultural, environmental, and behavioral factors. Their causality is thus both complex and multilevel. Many of the sociocultural factors influencing the development, spread, and persistence of chronic diseases are tied to macro-level factors that may include socioeconomic variables such as economic status, race, social status, education, and income. The wide variation in these factors adds to the complexity of public health efforts to address chronic disease globally. The articles published in this issue of *PCD* demonstrate the numerous contexts in which public health specialists throughout the world address chronic diseases and their causes and illustrate the wide variety of methods that they are using to do so.

The articles in this issue are like appetizers on the menu of a restaurant offering a plethora of choices. They represent just some of the possibilities. Strategic approaches to chronic disease prevention and health promotion generally fall into one of four categories: a community-based approach, a disease-based approach, a population-based approach, and a settings-based approach. Ideally, we would like to combine these approaches in order to address chronic disease in a more comprehensive fashion; however, in the real world of public health practice, which usually involves limited resources, this is not always possible. The articles in this issue also reflect the diverse methodologies that have become accepted practice in chronic disease prevention and health promotion efforts throughout the world. Thus we see articles that describe qualitative studies, descriptive studies, and case studies, as well as the use of focus groups, surveys, and surveillance. For example, the article by Mier et al on type 2 diabetes illustrates how chronic disease problems cut across international borders and how the particular context of an at-risk population needs to be taken into account if interventions are to be effective (2); the article by Minh et al shows the importance of a point-in-time survey to reveal the burden of chronic diseases in a rapidly developing country (3); O'Hegarty et al use focus group results to argue for policy change in cigarette labeling requirements by comparing the responses of adolescents to cigarette labels from two neighboring countries with quite different policies (4); Robinson et al use nearly two decades of efforts to address cardiovascular diseases in Canada as background to highlight the need for prevention programs to be more comprehensive and partnership oriented (5); and Ebrahim et al stress the need to more widely distribute information and interventions that address the behavioral risk factors related to chronic diseases; in doing so, they clearly show the need for systematic and timely surveillance of risk factors across the globe, a goal whose achievement unfortunately appears to be well into the future (6).

In all likelihood, chronic diseases will be the predominant global source of morbidity, death, and disease during the 21st century. Although much of the global community is benefiting from the accomplishments of medicine and public health, these benefits remain unevenly distributed. Studies and interventions such as those reported in this issue may hold part of the key to translating the successes of industrialized countries to countries that are less economically developed. Nevertheless, the science of public health is still underdeveloped in much of the world: chronic disease surveillance systems are spotty, behavioral risk surveillance is uncommon, and health promotion infrastructure is often lacking. To address the great burden of chronic disease globally, we will need significantly more studies and interventions of the type described here.
